# Impact of nitrogen on ectomycorrhizal fungi: from resource exchange to forest carbon cycling

**DOI:** 10.1093/jxb/erag010

**Published:** 2026-02-06

**Authors:** Filipa Cox

**Affiliations:** Department of Earth and Environmental Sciences, The University of Manchester, Manchester M13 9PT, UK; North Central College, USA

**Keywords:** Biogeochemical cycling, carbon allocation, carbon sequestration, ectomycorrhizal exploration strategy, ectomycorrhizal fungi, fungal community shifts, inorganic nitrogen, resource exchange, source–sink dynamics

## Abstract

Inorganic nitrogen (N) is known to influence the composition and functioning of ectomycorrhizal (ECM) fungal communities. Research consistently highlights fungal traits related to carbon (C) use as key determinants of fungal sensitivity to elevated inorganic N, with more C-demanding ECM fungi declining along inorganic N gradients. This decline is often attributed to reduced C allocation from host trees to their fungal symbionts, yet the precise mechanisms underlying this reduction remain unclear, despite significant research efforts. Here, recent advances in this field are examined, highlighting the role of fungal nutritional requirements and source–sink dynamics in regulating C flow to ECM fungi. Additionally, how N-induced shifts in ECM fungal communities impact biogeochemical cycles, potentially leading to globally significant changes in decomposition and C-sequestration rates in forest soils, is explored. Given the scale of these potential effects, further research is essential to fully understand the complexity of N-driven changes in ECM fungal functioning

## Teaser

A discussion of recent advances in how resource exchange between plants and ectomycorrhizas can drive responses of ectomycorrhizal fungal communities to nitrogen availability, along with the effects on forest carbon cycling.

## Introduction

Ectomycorrhizal (ECM) fungi are the predominant root symbionts in temperate and boreal forests, ecosystems historically limited by nitrogen (N) availability. In these environments, ECM fungi provide host plants with an enhanced source of N in exchange for host-derived carbon (C). It is an exchange of resources that is, despite decades of research, still not well defined in terms of what regulates the quantities moving between partners or its environmental dependency. Anthropogenic activities have roughly doubled the amount of bioavailable N entering the atmosphere and being deposited onto terrestrial surfaces (Ackerman *et al*., 2019), driving shifts in ECM fungal communities, likely by disrupting the flow of resources between hosts and their associated fungal communities. However, the precise mechanisms behind these effects are still uncertain, and may be driven by active regulation of C provisioning by the plant, host immunological responses, source–sink dynamics of C and N, and/or fungal influence via altered plant physiology. Understanding these potential mechanisms is important, because it has significant impacts on ECM fungal functioning and their critical roles in forest biogeochemical cycling, where they have the potential to enhance C sequestration or deplete C stores on a globally significant scale. This review examines recent work that sheds light on how inorganic N availability influences the dynamics of C exchange in ECM fungal–plant systems, improving our understanding of why ECM fungal communities change in response to N inputs. I then go on to consider the broader implications for forest biogeochemical cycling, highlighting new work on this important topic, as well as areas of uncertainty that require further study. Some of these key studies are summarized in Box 1.

Box 1. **Key developments**

Pena *et al*. (2023) Using dual labelling of stable isotopes of C and N to measure allocation between beech trees and their associated ectomycorrhizal (ECM) fungal communities, the authors demonstrated that C allocation declined with increasing C/N ratio of the ECM root-tip and that ECM fungal species receiving more C occupied more root-tips

Chen *et al*. (2024) Pure culture studies demonstrated that ECM fungal decomposition of soil organic matter was driven by fungal nutritional needs for carbon at both high levels of inorganic N availability and when in competition with another species. Additionally, ECM fungal species grown together demonstrated facilitation between more and less capable decomposers.

Choreño-Parra and Treseder (2024) A meta-analysis revealed that presence of ECM fungi enhanced rates of soil decomposition via priming effects at high fertility sites, whilst at low fertility sites their presence had no discernible effect on decomposition, indicating that Gadgil effects may be less significant than previously thought, relative to priming.

Jörgensen *et al*. (2024) An investigation into ECM fungal communities across natural fertility gradients differing in rates of inorganic N deposition, showing that ECM fungal communities remained relatively stable across natural gradients but were sensitive to external inputs of inorganic N, particularly at the highest natural fertility sites.

## Nitrogen availability alters ectomycorrhizal communities

Soil environments are inherently heterogeneous, and ECM fungi compete for N in a huge diversity of forms. Organic N, which represents about ∼95% of total soil N, ranges from simple monomers to complex macromolecules, each varying in chemical composition and recalcitrance (Talbot and Treseder, 2010). Coupled with the high diversity of ECM fungi—over 20 000 species, which have independently transitioned from saprotrophic ancestors perhaps ∼80 times (Tedersoo and Smith, 2017)—there is strong potential for niche partitioning of N among co-occurring fungi (Tedersoo *et al*., 2003). This hypothesis is supported by differential retention of genes for soil organic matter decomposition from saprotrophic ancestors across fungal lineages (Kohler *et al*., 2015), and from pure culture studies that indicate marked differences in the ability to access N sources of increased complexity across fungal taxa (Nygren *et al*., 2008; Chen *et al*., 2024). Given their primary role in supplying N to host plants and this strong gradient of access to different N sources, it is perhaps unsurprising that ECM fungi can be highly sensitive to increased N availability. Indeed, numerous studies have demonstrated that N is a key factor structuring their communities at multiple levels, including overall abundance and biomass, as well as shifts in community composition and, sometimes, species richness (Cox *et al*., 2010; Kjøller *et al*., 2012; van der Linde *et al*., 2018; Jörgensen *et al*., 2022).

In N-poor boreal forest soils where fungi are themselves N limited, ECM fungal biomass and hyphal production can increase along natural N gradients, at least to a point (Sterkenburg *et al*., 2015; Högberg *et al*., 2021), whilst declines relative to other fungal guilds are often observed at highest N fertility (Högberg *et al*., 2006; Mayer *et al*., 2021; Pellitier and Zak, 2021). Anthropogenic inputs of inorganic N frequently appears to drive declines in ECM fungal biomass and relative abundance across a range of spatial scales, although strength of response can depend on the natural background fertility of the forest (Bahr *et al*., 2013; Högberg *et al*., 2014; Guo *et al*., 2021; Jörgensen *et al*., 2022). These sometimes inconsistent biomass responses to N were recently explored using two natural N gradients, one of which was subject to additional N inputs via deposition (Jörgensen *et al*., 2024). This study found that ECM fungal biomass and composition remained relatively stable across natural N gradients, while N deposition reduced biomass and induced community shifts, even though inorganic N levels were similar in both cases. This suggests differential responses to N derived from anthropogenic inputs as opposed to N resulting from endogenous processes, although the sensitivity of ECM fungal communities to additional N inputs via deposition increased at higher natural fertility levels. These findings highlight how different forms of N, and the environmental context of the forest, alter responses of ECM fungi.

Responses to external inputs of additional N often manifest as distinct shifts in ECM fungal community composition, allowing many species to be confidently categorized as ‘nitrophilic’ or ‘nitrophobic’ (van der Linde *et al*., 2018). These two groups often, but not always, differ in a number of morphological and functional traits relating to host preference and N and C use (Lilleskov *et al*., 2011, 2019). For example, species that decline in abundance with increased inorganic N availability are often specialized to coniferous forests and possess greater enzymatic capabilities to decompose recalcitrant organic matter, whilst those increasing in abundance are typically associated with broadleaf trees or exhibit non-specific host preferences (van der Linde *et al.,* 2018). Several studies have also noted that the exploration types of ECM fungi (Agerer, 2001), characterized by their mycelial growth patterns and biomass accumulation, also exhibit varying responses to N enrichment (Lilleskov *et al*., 2011; Suz *et al*., 2014; Pellitier and Zak, 2021). For instance, high biomass ‘medium-distance fringe’ types (described as forming extensive external mycelia with abundant hydrophobic cords and rhizomorphs) tend to decline under elevated N, while lower biomass ‘contact’ types (forming smooth hydrophilic mantles with very sparce external mycelia) can increase in abundance (Suz *et al*., 2014). However, recently it has been proposed that current definitions of exploration type do not correlate consistently with responses to N availability (Jörgensen *et al*., 2023). Instead, the authors suggest that fungal growth-rate could be a better predictor of responses to inorganic N, with slow-growing, hydrophobic fungi being typically nitrophobic, and fast-growing species nitrophilic. Such physiological differences between nitrophobic and nitrophilic ECM fungal species might be suggestive of inherent differences in N use strategy and C use efficiency (CUE) along a trait spectrum. Jörgensen *et al*. (2025) propose that fast-growing fungi are typically N ‘absorbers’ adapted for uptake of inorganic N and able to convert more host-derived C into growth versus other pathways such as production of costly enzymes, whilst slow-growing fungi are N ‘miners’ capable of accessing more complex N, but with a resultant lowering of CUE.

Whether or not nitrophobic fungi have greater enzymatic production, higher biomass, and/or slower growth rates compared with their nitrophilic counterparts, the general pattern appears to be that ECM fungi with higher C requirements decline in abundance with elevated inputs of inorganic N. This overarching theme provides a mechanistic link to the observed changes in both ECM fungal biomass and community structure. The prevailing view is that this apparent trend is driven by lower C supply from hosts in response to elevated N—when plants experience a reduction in N limitation, the proportion of C allocated belowground to roots and ECM fungi is reduced in favour of aboveground parts (Högberg *et al*., 2010; Eastman *et al*., 2021). Studies have indicated that reduced C allocation belowground can have a disproportionate effect on nitrophobic ECM fungi. For example, the nitrophobic genus *Suillus*, known for its high mycelial biomass, shows reduced colonization under decreased C supply, such as following defoliation of host *Pinus sylvestris* (Kuikka *et al*., 2003). The presence of strongly nitrophobic *Cortinarius* spp. has been linked to low CUE of fungi in Swedish forest soils (Hagenbo *et al*., 2019), likely due to the particularly high enzymatic activity of these fungi. While our understanding of the traits related to C-demand exhibited by nitrophilic and nitrophobic fungi is steadily growing, the precise mechanisms that result in reduced C allocation to ECM fungi with elevated N availability are not yet fully resolved (Bogar, 2024; Bunn *et al*., 2024) and I explore this further in the next part of this review.

## Mechanisms driving reduced C allocation to ectomycorrhizal fungi

### Regulation by host plants

Host plants may influence ECM fungal communities by allocating C preferentially to fungi that enhance nutrient acquisition, in line with ecological market trade theory (Kummel and Salant, 2006). Under low inorganic N availability, the optimal fungal partners are likely those that provide greatest access to complex organic N (i.e. ‘miners’ *sensu*  Jörgensen *et al*., 2025), so under these conditions the plant may facilitate greater occurrence of fungi with enhanced enzymatic abilities (and greater C requirements). Under conditions of elevated inorganic N, plant requirement for ECM fungal species with enhanced enzymatic capability reduces, and the optimal partner therefore becomes the least C-demanding species that might specialize in inorganic N uptake (i.e. ‘absorbers’ *sensu*  Jörgensen *et al*., 2025) or offer other host benefits. In arbuscular mycorrhizal systems, plants have been observed to direct more C to beneficial fungal partners (Kiers *et al*., 2011). However, in the ECM symbiosis evidence for such selective C allocation is mixed. There is sometimes a positive correlation found between the amount of N the fungus provides to the plant and the amount of C provided to the fungus (Bogar *et al*., 2019), but the results can be highly dependent both on the identity of the ECM fungus and on environmental conditions such as N availability and competition (Bogar *et al*., 2022). Other studies have shown no correlation between the N and C exchanged (Hortal *et al*., 2017; Plett *et al*., 2020; Horning *et al*., 2023), or even negative correlations where less N was provided as C allocation increased (Hasselquist *et al*., 2016).

One mechanism by which plants could potentially control provisioning of C is via regulation of invertases extruded into the mycorrhizal interface, which cleave plant-derived sucrose into fungal accessible fructose and glucose (Nehls *et al*., 2010). However, the extent to which this process could promote or restrict C to particular fungal symbionts is not currently known, and some evidence suggests that the availability of root hexoses does not always impact on mycorrhization, at least in AM systems (Schaarschmidt *et al*., 2007). In addition, some ECM plants do not appear to be C limited (Corrêa *et al*., 2012) so do not experience a C cost in supporting their symbiotic partners, or indeed need to restrict C-transfer from the perspective of saving resources. In summary, evidence for market-trading of resources is complicated, may only operate in certain scenarios, and does not appear to provide an adequate explanation behind reduced C flow to ECM fungi under elevated N. Further studies aimed at assessing a broader suite of potential ECM fungal benefits outside of N provisioning, such as water, P, and micronutrient uptake, as well as pathogen resistance, should provide greater insight into the relative importance of resource trading in ECM symbioses.

An alternative mechanism by which plants could actively influence the identity of their ECM fungal symbionts is via up-regulation of defence genes to limit degree of colonization. Hortal *et al*. (2017) found that when colonized by an ECM fungal isolate that transferred less N to its host plant than competitors, plants up-regulated defence gene transcription and reduced ECM formation by the less beneficial fungus. Recently, Stuart *et al*. (2023) found that plant C transfer to various strains of *Pisolithus microcarpus* was explained less by the proportion of root-tips colonized than by the expression of genes related to plant defence and stress responses. Thus, there is some evidence that host plant defence responses may by a primary pathway by which plants can regulate the species with which ECMs are formed. However, there is a relative paucity of work investigating the importance of these plant immune responses, which are likely to be highly species- and context-specific, in regulating C flow into mycorrhizas.

### Stoichiometry and source–sink dynamics

Beyond plant-driven selection of optimal fungal partners, simple source–sink dynamics and fungal tissue stoichiometry could also play an important role in shaping ECM fungal communities across N availability gradients. This differs from ecological market trade theory in that instead of the reciprocal exchange of resources being trade-based—that is, that higher fungal provisioning of N is rewarded by greater C allocation by the plant—it is instead driven by the relative C sink strength of the partners. Recent pure-culture studies indicated that N availability alters fungal requirement for C—under elevated inorganic N, ECM fungi decompose C compounds in soil organic matter (SOM) to a greater extent than under low N conditions, in order to satisfy their increased C demands (Chen *et al*., 2024). This effect was more pronounced in species with greater ability to decompose SOM and higher production of extrametrical mycelium. When ECM fungi are in symbiosis with a plant, the prevailing view is that they gain all their C from plant photosynthate (Lindahl and Tunlid, 2015; Kuyper, 2017). If fungal C demand increases with elevated N, fungi with higher C requirements may become more C limited if the plant does not—or cannot—supply sufficient C to its fungal partner. This scenario is exacerbated by the tree’s own response to increasing N availability where allocation of C to roots decreases (Eastman *et al*., 2021; Marshall *et al*., 2023). The net effect of higher fungal C requirements and reduced host C supply under elevated inorganic N may result in the persistence of only those ECM fungal species capable of surviving on limited C supply.

If simple source–sink dynamics are a major driver of resource exchange, we might then expect that under N-limited conditions, ECM fungal species with greater enzymatic potential can access complex organic N sources, potentially lowering their internal C:N ratios and creating a stronger C sink. A fungal-driven C:N ratio driving C allocation from the plant has been recently demonstrated in young beech trees, where ECMs with higher C:N ratios were allocated less C than ECM fungal species with lower C:N ratios (Pena *et al*., 2023). This additional C allocation was further correlated with the proportion of root tips colonized by the same ECM fungal species, suggesting that it enhanced fungal fitness. In this way, more C-demanding ECM fungal species can be maintained in an ecosystem as their better N mining activities are ‘rewarded’ with additional C as a response to their lower C:N ratio and greater C sink strength. In natural N gradients with low N mineralization rates, such as the boreal forest studied in Jörgensen *et al*. (2024), co-limitation of fungal C and N may preserve these source–sink dynamics, with this dynamic only becoming disrupted when there are inputs of external N that enable trees to directly take up inorganic N (Castro-Rodríguez *et al*., 2017), bypassing their fungal symbiont (Zhang *et al*., 2019; Jörgensen *et al*., 2024).

Beyond simple source–sink dynamics, there is also the potential for the fungal symbiont to increase its C supply through influence on plant physiology and its own C sink strength. In this case, the most successful fungal partners might not necessarily be the most beneficial in terms of nutrient provision, but rather be the most effective as manipulators of their hosts. For example, by prioritizing retention of N for fungal growth and biomass accumulation instead of provisioning host plants, ECM fungi can drive increased C allocation from trees under N-limited conditions (Näsholm *et al*., 2013; Hasselquist *et al*., 2016). Additionally, there is potential for fungal-driven influence of plant physiology. Ectomycorrhizal fungi can produce a number of plant growth regulating compounds that induce morphological changes in roots. For example, the ECM fungus *Laccaria bicolor* can produce and secrete auxins, a class of plant hormones. These fungal-derived auxins have been shown to stimulate the formation of lateral roots of ECM plants (Vayssières *et al*., 2015). By actively remodelling the host’s root architecture, the fungal partner increases the surface area available for colonization, thereby creating a larger potential sink for the plant’s C resources and increasing its own fitness.

Fungal C sink strength may change in response to prevailing environmental conditions. For example, in competitive situations, ECM fungi may represent more of a C-sink, as they require additional C to prioritize hyphal production to dominate space (Chen *et al*., 2024). Global climate drivers such as precipitation and temperature may also alter source–sink dynamics. For example, in a boreal–temperate forest system, warming and reduced rainfall generally caused shifts in ECM fungal communities towards low biomass ‘contact-short’ exploration types (Fernandez *et al*., 2023). However, certain C-costly long-distance exploration Suilloid species increased in abundance, potentially because their hydrophobic rhizomorphs could promote water transport under low moisture conditions (Fernandez *et al*., 2023), or alternatively because these taxa are good at acquiring host-derived C even when in short supply (Bruns *et al*., 2002).

In summary, considerable work has revealed several potential routes through which C supply can be regulated to ECM fungi, including both control by plant and fungal partners and source–sink dynamics. The likelihood is that none of these mechanisms operates independently, and the picture is probably complex, especially considering the huge diversity of ECM fungi, plants, and environmental contexts, which may all elicit differential controls on the exchange of resources. Nonetheless, more recent studies are, perhaps, beginning to suggest that market-trade driven exchanges are less important in regulating C allocation to ECM fungi, whilst fungal-driven resource requirements and resultant sinks for C might be more important. Future work will help narrow down the dominant pathways across environments, but functionally, the shifts in ECM fungal communities away from ECM fungi with typically nitrophobic traits towards nitrophilic ones will have substantial and complex implications for forest biogeochemical cycling, which I explore next.

## Consequences for forest biogeochemical cycling

Soil contains a huge potential C source or sink, with ECM fungi playing pivotal roles in its dynamic fluxes. They act as conduits of photosynthetically derived C belowground (Högberg *et al*., 2025), directly (Shah *et al,* 2016; Lindahl *et al*., 2021; Chen *et al*., 2024) and indirectly (Choreño-Parra and Treseder, 2024) contribute to SOM decomposition, and serve as substantial C reservoirs through their live and dead hyphae (Hawkins *et al*., 2023; Hagenbo *et al*., 2024). The presence of ECM fungi has been shown to both accelerate (Choreño-Parra and Treseder, 2024) and decelerate decomposition of SOM (Averill and Hawkes, 2016), with the direction and magnitude of the effect often dependent on factors such as site-specific N availability and the soil’s C:N ratio (Argiroff *et al*., 2022; Mayer *et al*., 2023; Choreño-Parra and Treseder, 2024). Given the globally significant C stocks sequestered in soils and the complex interactions between N availability, ECM fungal communities, and C cycling, much recent research has focussed on elucidating the links and drivers between these interrelated components of forest biogeochemical cycling. The main pathways by which N-induced shifts in ECM fungal communities might influence soil C-cycling are shown in Box 2.

Box 2. **Effects of plant–mycorrhizal resource exchange driven shifts in ectomycorrhizal fungal communities on soil biogeochemical cycling**

Increasing inputs of inorganic N drive ectomycorrhizal (ECM) fungal communities from nitrophobic to nitrophilic taxa, with concurrent shifts in ECM traits related to C and N usage and their influence on forest biogeochemical cycling. Under low inorganic N availability, mineralization rates are low, and trees are dependent on ECM fungi to take up almost all of their N from organic N sources. Under these conditions, plant C allocation to ECM fungi is high. This promotes ECM fungal abundance and activity, increasing abundance of C-demanding species with enhanced decomposer capabilities and increasing ECM fungal decomposition of soil organic matter (SOM). However, increased ECM fungal uptake of N inhibits saprotrophic decomposition of SOM, and overall rates of decomposition may be supressed. At high levels of inorganic N, plants bypass ECM fungi to uptake N directly, and C flow to ECMs is reduced. Low C-demanding ECM fungi persist whilst high C demanding species decline in abundance. Lower ECM fungal activity results in reduced hyphal biomass and decomposer activity. However, inhibition of free-living saprotrophs is reduced, and ECM fungi play a bigger role in priming saprotrophic decomposition of SOM. Although uncertain at this point, there is evidence that there is increased formation of mineral-associated organic matter as ECM fungal communities shift in response to elevated N, increasing long-term C storage potential. Figure created in BioRender. Cox, F. (2025) https://BioRender.com/9o3dhnx

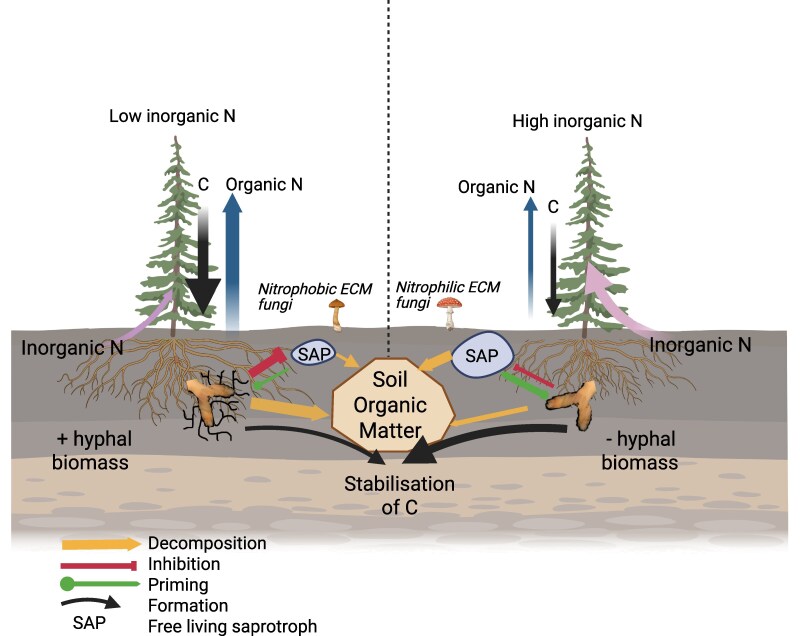

### Direct impacts of ectomycorrhizal fungi across N gradients

Ectomycorrhizal community compositional shifts induced by inorganic N availability likely have direct implications for forest biogeochemical cycling. Several studies have reported that ECM fungal taxa dominant under low inorganic N availability typically exhibit high SOM decomposition capacity, whereas those with limited decomposition capacity tend to prevail under high inorganic N conditions (Argiroff *et al*., 2022; Mayer *et al*., 2023). Abundances of certain ECM fungi with greater enzymatic potential may be linked to a reduction in C storage or longer-term decomposition of more recalcitrant humus (Sterkenburg *et al*., 2018; Lindahl *et al*., 2021). In pure culture conditions, these enzymatically capable ECM fungi up-regulate decomposition of SOM under conditions of increased inorganic N availability, targeting the C compounds in SOM to meet their own C demands (Chen *et al*., 2024). In natural systems, these increased C demands might be met by the plant, although overall reduced C allocation belowground with elevated inorganic N could prevent this. Whilst the prevailing view is that ECM fungi do not utilize the C in SOM when in symbiosis, there may be environmental contexts where ECM fungi from particular lineages can engage in facultative saprotrophy, potentially up-regulating SOM decomposition and altering the SOM compounds targeted to gain a supplementary source of C. A recent study demonstrated that organic N induced the facultative ECM fungus *Clitopilus hobsonii* to form a symbiosis on the roots of *Populus tomentosa*, but when availability of inorganic N was high, the mutualism was disrupted and saprotrophy was promoted (Peng *et al*., 2022). This study gives insights into how some fungal species could maintain dual trophic status, potentially switching functional guilds depending on the availability of different forms of N. While such clear functional examples are still relatively rare, genomic studies suggest the potential for saprotrophy may be more common, as many ECM fungi have retained genes for degrading complex organic matter (Miyauchi *et al*., 2020). However, the ubiquity and ecological significance of this potential ‘trophic flexibility’ in natural systems is not yet known.

Exploring how N availability influences ECM fungal induced decomposition across soil horizons is an emerging area of research. Mayer *et al*. (2023) observed that ECM fungi enhanced SOM decomposition at high levels of N availability but reduced SOM decomposition at low levels of N. However, these responses were limited to the mineral topsoil, whilst no response was observed in the organic layers. Furthermore, a recent study indicated that mycorrhizal weathering of silicates in the mineral horizon is intrinsically linked to sufficient N provided by the organic horizon (Mahmood *et al*., 2024), but the authors predict that this stimulation of weathering would not occur with inorganic N input. Understanding how external inputs of N might disrupt links between natural fertility gradients and ECM fungal decomposition across soil profiles requires further study, as C held in deeper soil layers is generally older and more resistant to decomposition, providing longer term C sequestration potential (Kirschbaum *et al*., 2021).

Ectomycorrhizal fungi have the potential to play a significant role in global soil C sequestration via numerous pathways including the biogeochemical recalcitrance of their hyphae and their significant influence on soil C stabilization via the promotion of mineral–organic associations. Trees allocate approximately 13% of their net primary productivity to ECM fungi (Hawkins *et al*., 2023) and their extraradical hyphae can constitute up to half of the C stored in soils (Clemmensen *et al*., 2013). Their extensive colonization of the soil environment provides a physical pathway for the movement of rhizospheric C to mineral surfaces, enabling the formation of mineral-associated organic matter (MAOM) (See *et al*., 2022), which can persist for extended periods, ranging from decades to millennia (Schmidt *et al*., 2011; Dungait *et al*., 2012). Nitrogen addition can influence the direction and magnitude of MAOM formation and the relative importance of hyphae versus root-derived residues. In ECM-dominated alpine forest systems subjected to chronic N addition, fungal hyphae enhanced soil C stabilization compared with plant roots, via increases in microbial-derived C and a greater potential for physical and chemical binding to mineral surfaces (Zhu *et al*., 2022). Hicks Pries *et al* (2023) found evidence that the community composition of ECM fungi was linked to the extent of MAOM formation, with decreasing MAOM occurring where the dominant ECM fungi combined key physical traits, such as medium-distance exploration types and more melanized hyphae, with a greater biochemical potential to produce peroxidases, suggesting that inorganic N-induced shifts in ECM fungal communities could increase the MAOM formation potential of the community.

Melanized fungal hyphae, such as those found in ECM fungal species like *Cenoccocum geophilum*, are notably resistant to degradation, allowing particulate matter to persist in litter bags for years (Fernandez *et al*., 2019). Fungal mycelial chemistry, including the degree of melanin content, can vary considerably among fungal species (Fernandez and Kennedy, 2018). Recently, it was demonstrated that anthropogenic N addition caused a reduction in late-stage decomposition of fungal necromass, with decomposition rates additionally driven by hyphal chemistry (DeLancey *et al*., 2025). Together this suggests that shifts in ECM fungal composition and subsequent changes in community level hyphal chemistry in response to elevated N, as well as the availability of N itself, has the potential to alter forest C and N release. Given the huge C pool that fungal mycelium represents (Clemmensen *et al*., 2013; Hawkins *et al*., 2023), these effects are likely to be globally significant, although the direction and magnitude of change are not yet well understood and likely to be highly context specific.

### Indirect effects on decomposition

#### Facilitation and microbial associations

Ectomycorrhizal fungi can alter the composition and activities of microbial communities in the mycorrhizosphere, promoting or inhibiting decomposition of SOM indirectly. Ectomycorrhizal fungal exudation of photosynthetically derived labile C can stimulate decomposition in C-limited microorganisms, whilst the ECM fungi may benefit from the N released. Additionally, the decomposition of SOM by ECM fungi themselves can depolymerize C, creating a more labile source for other microorganisms to utilize (Shah *et al*., 2016). This effect was demonstrated in pure culture, when ECM fungi capable of decomposition were grown on SOM extracts alongside ECM fungi with poor decomposer ability. In the absence of the capable decomposer, poor decomposers could not grow. However, in the presence of the capable decomposer they grew well by utilizing the labile resources released from decomposition, suggesting facilitation between the species (Chen *et al*., 2024). Similar scenarios in natural systems may give rise to co-occurrence of ECM fungal taxa, as well as other functional guilds, across root-tips and soil.

The remarkable diversity in the morphology and physiology of ECM fungi creates unique microenvironments for soil microorganisms. This includes the potential that they modify exudates released from roots via mycorrhizal re-uptake of root-derived N, altering the C:N ratio of resources available to rhizosphere microbial communities, which may happen in a taxon-specific manner (Pena *et al*., 2023; Pena and Tibbett, 2024). In beech forests, root-tips from different ECM fungal species were found to be associated with different microbial communities, supporting taxonomically distinct communities of bacteria, archaea, and other fungi (Dietrich *et al*., 2022). Interestingly, both dominant and rare ECM fungal species significantly influence the composition of these microbial communities. Members of the microbial communities included bacteria known to assist in ECM fungal formation, raising the possibility of bacterial-mediated positive feedback loops as specific bacteria can promote ECM formation by specific fungal taxa (Reis *et al*., 2021). Additionally, ECM fungal necromass serves as a habitat for diverse bacterial and fungal decomposers, with community structures influenced by the underlying chemistry of the dead hyphae (Beidler *et al*., 2020; Cantoran *et al*., 2023). Species-specific ECM microbiomes suggest the potential for ECM-fungal-driven shifts in associated microbial communities as ECM fungal communities change across environmental gradients such as N availability. Reductions in abundance of ECM fungi have recently been linked to sharp declines in ectomycorrhizal helper bacterial communities over time (Berrios and Peay, 2025).

#### Antagonistic interactions and the Gadgil effect

There may also be effects on decomposition rates via the Gadgil effect—a long-standing hypothesis suggesting antagonistic interactions between saprotrophic and ECM fungi (Gadgil and Gadgil, 1975). The proposed mechanism is driven by ECM fungal immobilization of N in their biomass and subsequent N limitation of saprotrophic decomposition, though the general relevance of this effect has been debated for some time (Fernandez and Kennedy, 2016). Fernandez *et al*. (2020) demonstrated that Gadgil-related inhibition of decomposition was only present where there was a high C:N ratio of litter and the ECM fungal community was enzymatically equipped to break down this litter—that is, in tannin rich forest floors typical under conifers. This pattern was also observed across natural fertility gradients in a temperate beech forest, where high abundance of ECM fungi inhibited saprotrophic activity at low fertility sites, but contributed to overall increased rates of decomposition at high fertility sites (Mayer *et al*., 2023). However, a reduction in Gadgil effects was not observed in a long-term fertilization experiment in northern Sweden (Maaroufi *et al*., 2019). Indeed, the overall importance of the Gadgil effect as a major driver of C sequestration at a global scale has been questioned with a recent large-scale meta-analysis concluding that its significance was highly context dependent (Choreño-Parra and Treseder, 2024).

To summarize, shifts induced in ECM fungal communities at high inorganic N have the potential to drive substantial shifts in forest biogeochemical cycling with consequences for the globally significant stores of C held in forest soils. While this area is gaining increasing research attention, further studies are needed to understand how N-induced changes in ECM fungal communities, and their intimately associated microbiomes, affect C cycling across soil depths and C pools of differing stability.

## Overall conclusions and future directions

There is broad agreement that the ECM fungal taxa most sensitive to inorganic N inputs typically have high C requirements, and that their decline is largely driven by reduced plant C allocation as inorganic N availability increases. However, the mechanisms regulating C flow from plants to ECM fungal symbionts—and their relative importance across different environmental contexts—remains unclear. Evidence for resource trading as a primary mechanism is mixed, but that evidence is primarily based on a narrow definition of fungal benefit (i.e. N provisioning) and does not consider wider advantages of the symbiosis. Further studies that include a broader definition of fungal benefit, including dimensions such as phosphorus uptake, pathogen resistance, and water provision across a range of environmental gradients may provide new insights.

Instead of a trade-based exchange of resources, recent research indicates that fungal C:N stoichiometry and C sink strength may play a dominant role driving movement of C and N between host plant and fungal partners, potentially interacting with active plant regulation via host immunity under certain conditions. Investigating the role of source–sink dynamics in C allocation between plants and ECM fungi opens up several avenues for future research. One key area is exploring whether different taxonomic lineages of ECM fungi have systematic differences in their tissue stoichiometry, or the degree to which they can tolerate a range of stoichiometric values in response to environmental conditions. Refining how fungal C sinks change in response to environmental conditions will also provide further insights. For example, competition appears to change the C-sink strength of fungi in controlled lab settings, but we need further work to assess the ecological relevance of this in complex natural systems. Furthermore, how N availability and climate gradients—as well as interactions between them—alter source–sink dynamics, will shed light on how this process operates across environmental contexts, as well as the tolerance levels of various ECM fungal taxa to fluctuations in N or C availability.

The functional consequences of shifts in ECM fungal communities in response to elevated inorganic N availability is potentially far-reaching, impacting on numerous aspects of forest biogeochemical cycling. One important area of future research is studies aimed at assessing how shifts in dominant ECM fungal taxa from ‘miners’ to ‘absorbers’, and their associated traits, might change C storage. These studies could focus on their role in SOM decomposition, the recalcitrance of their hyphae, and the extent to which particulate organic matter or MAOM fractions are formed across soil horizons. Lastly, how ECM fungal traits interact to drive other key microbial communities, including the diverse suite of microorganisms that live on ECM root-tips and external mycelia, is key to gaining a more holistic view of how ECM fungal community shifts in response to N will alter wider microbial-driven processes.
